# Improving Colorectal Screening Compliance Through Targeted Quality Improvement Interventions in Primary Care

**DOI:** 10.7759/cureus.96031

**Published:** 2025-11-03

**Authors:** Olubunmi Oladunjoye, Isabel Valdez

**Affiliations:** 1 Section of General Internal Medicine, Baylor College of Medicine, Houston, USA

**Keywords:** colorectal cancer, improvement, outcomes, quality, screening

## Abstract

Background: Colorectal cancer (CRC) is the third most common cancer in the United States, and yet it is one of the most preventable cancers. Despite multiple available screening options, national CRC screening rates remain suboptimal. There is an observed* *lower participation among middle-aged adults compared with those 65 years and older. Therefore, targeted interventions to improve CRC screening in those younger than 65 years old are needed.

Objective: Increase CRC screening rates by 10% among patients aged 45-64 years old in a primary care clinic between July 2023 and May 2024.

Methods: A multimodal intervention approach that utilized (1) educational presentations for faculty and nursing staff on processes, indications, risks, and benefits of both invasive and non-invasive CRC screening methods; (2) development and dissemination of educational patient brochures for patients in the clinic; and (3) delivery of electronic patient portal messages containing education on risks of colon cancer and screening modalities to all 2,020 patients who were overdue for CRC screening or had never completed screening. Subsequent automated reminders were also sent on days 15, 30, and 45. The main measure was the monthly CRC screening rate among patients aged 45-64 years old.

Results: CRC screening rates increased from 65.5 to 74.3% over a 12-month period. A more consistent upward trend was seen after Plan-Do-Study-Act (PDSA) cycle 3, which incorporated patient portal messaging and automated reminders.

Conclusion: Implementation of multimodal interventions that include clinician training, patient education, and targeted patient outreach can bring about a positive impact on CRC screening rates. While temporal association suggests these interventions may have contributed to improvements, more rigorous study designs are needed to establish causation.

## Introduction

Colorectal cancer (CRC) is the third most common cancer in the United States and is one of the most preventable cancers [1, 2]. Multiple screening methods exist, and although screening rates have increased, the national rate remains under 80% (a national goal set by the National Colorectal Cancer Roundtable, which was endorsed by the American Cancer Society) [3]. Additionally, the high incidence and mortality of this cancer have affected a younger population, leading the United States Preventive Services Task Force (USPSTF) to lower the CRC initial screening age from 50 years old to 45 [4, 5]. However, screening rates remain lower among those aged 50-64 years old when compared with those older than 65 years old. According to the American College of Gastroenterology, CRC screening rates are 57.9% among adults aged 50-64 years, compared to approximately 80% among those aged 65 years and older, indicating a disparity of roughly 20% between the two age groups. The rate of screening was estimated to be about 34% in those aged 45-49 years [1, 6]. 

Several barriers to CRC screening have been reported, and the key to increasing screening rates requires improved understanding of the barriers to screening and methods to circumvent them. These include lack of patient knowledge and health literacy about CRC screening, persistence of myths about CRC, fear or discomfort with the procedure, bowel preparation issues, socioeconomic factors (e.g., procedure cost, time off work), and logistical challenges like transportation [7, 8]. A major challenge identified in our clinic was that patients presenting with multiple comorbidities or complex clinical concerns often had limited time during visits to address preventive care measures, including screening reminders. Clinicians also face barriers, including a lack of knowledge about high-risk patients, insufficient screening recommendations, and limited time to educate patients [9, 10]. However, studies show providers are more likely to discuss screening when they receive point-of-care or electronic health record (EHR) reminders [7]. 

In a review by Jain et al., multiple screening strategies should be offered based on invasiveness, accessibility, and cost [11]. Inadomi et al. found that the most effective interventions for improving screening rates and addressing barriers include employing patient navigators, appropriate screening selection for patients, and outreach efforts like mailing or distributing stool-based CRC screening tests [12, 13]. They also showed that using multiple strategies enhances adherence, and offering a fecal blood test alone or as an option alongside colonoscopy led to higher adherence rates than offering only colonoscopy. 

In this quality improvement study, we aimed to increase CRC screening rates by 10% among patients aged 45 to 64 years old in our outpatient clinic over a one-year period. 

This article was previously presented as a meeting abstract at the 10^th^ Annual Quality Improvement and Patient Safety Conference, Baylor College of Medicine, Houston, TX, USA, on April 23, 2024.

## Materials and methods

A multimodal intervention approach was used for this study. This study was deemed exempt by the Institutional Review Board. We identified our target population as patients aged 45 to 64 years old who did not have an updated CRC screening documented in the EHR. A total of 2,020 patients were identified as eligible and due for screening. The first Plan-Do-Study-Act (PDSA) cycle was implemented in August 2023, cycle 2 in November 2023, and cycle 3 in February 2024. In our first PDSA cycle, we provided an educational presentation to our faculty and staff on the processes, indications, risks, and benefits of both invasive and non-invasive methods (fecal-based test) for CRC screening. During this presentation, we encouraged faculty to offer alternative screening methods for patients who declined colonoscopy. We also demonstrated the use of an existing real-time clinical decision support tool in our EHR that alerts the clinician to the patient’s screening care gaps and facilitates ordering. This tool has an easy-to-use set of orders for both colonoscopy and other noninvasive CRC screening tests embedded in the alert to facilitate ordering the screening test while reducing the work burden on the clinicians. The non-invasive CRC screening tests embedded in the alert include the multitarget stool DNA (mt-sDNA; Cologuard, Exact Sciences, Madison, WI, USA) and fecal immunochemical test (FIT). For the second PDSA cycle, educational patient brochures were designed and printed for distribution to patients in the clinic. These brochures educated the patients on CRC risks, the differences in the screening modalities offered in our institution, and clinic contact information. These brochures were prominently displayed in the clinic work area and exam rooms. In our final PDSA cycle, electronic messages were sent to all 2,020 patients via the secure message patient portal, and automated reminders were sent on days 15, 30, and 45 from the initial message. The message contained information about CRC and different screening methods, offering both the mt-sDNA CRC stool test and colonoscopy. Patients were encouraged to respond to the message with their preferred screening methods, colonoscopy or the mt-sDNA CRC stool test. After reviewing the patient’s response and medical records, orders were placed for the patient's preferred or appropriate screening modality based on their medical history or family history. If a patient had already completed their screening, steps were taken to update the medical records either by manually entering results sent by patients or updating the interval for screening recommended by their specialists or pathology results.

Our outcome measure was the percentage of patients aged 45-64 years who had documented completion of CRC screening in the EHR, including both newly completed screenings and updated documentation in the EHR of prior screenings conducted elsewhere as of June 2024. The data were obtained monthly from our quality data reporting team. Process measures included the percentage of persons who responded to the automated messages and the percentage of persons for whom CRC screening orders were placed. We also collected information on age, race, and ethnicity as self-reported by patients. Our study categorized ethnicity as Hispanic and non-Hispanic, consistent with how demographic data were captured in our EHR system.

## Results

A total of 2,020 patients were eligible for screening, and electronic messages were sent to all, with 1,919 of these patients having active MyChart accounts (Epic Systems Corporation, Verona, WI, USA) (95%). Of the 2020 patients, 904 (44.8%) were males, 975 (48.3%) self-identified as White, and 521 (25.8%) as Black or African American. Of those who had active MyChart accounts, 577 patients (30%) responded to the MyChart messages. There was no difference in the mean ages of those who did and did not respond. However, non-Hispanic patients were more likely to respond (p=0.006). Among White patients, 318 (33.5%) responded to the electronic message compared to 31.2% of the Black/African American patients (p<0.001) (Table 1). From the 577 respondents, 320 CRC screening orders were placed, including 244 mt-sDNA CRC stool tests (Cologuard), 74 colonoscopies, and two patients with both mt-sDNA CRC stool tests followed by colonoscopy orders. Among the 577 respondents, 25 patients (4.3%) declined screening, 28 patients (4.9%) reported they were no longer patients of our clinic, 135 patients (23.4%) had already completed screening (of which updated records were provided for 92, where screening dates were available), and 51 patients (8.8%) deferred to their primary care providers or gastroenterologists or had orders previously placed by their providers that had yet to be completed (Table 2).

**Table 1:** Sociodemographic Characteristics of the Patients *Statistical significance p<0.05; **Our study categorized ethnicity as Hispanic and non-Hispanic, consistent with how demographic data were captured in our electronic health record system.

Variable	Total number of patients due for screening; N (%) Total N=2020	Active portal (N=2020)			Responded to the message			Requested orders to be placed*		
		Inactive Portal N=101	Active Portal N=1919	Total N=2020	No N=1342	Yes N=577	Total N=1919	No N=257	Yes N=320	Total N=577
	N (%)	n(%)	n(%)	p-value*	n(%)	n(%)	p-value*			p-value*
Age, years (mean ± std))	53.9 ± 5.7	56.4 ±5.3	53.8 ±5.7	<0.001	53.7 ±5.7	53.8 ±5.8	0.619	54.6 ±5.4	53.2 ±6.0	0.001
Sex				0.044			0.053			0.383
Male	904 (44.8)	55 (6.1)	849 (93.9)		613 (72.2)	236 (27.8)		100 (42.4)	136 (57.6)	
Female	1116 (55.2)	46 (4.1)	1070 (95.9)		729 (68.1)	341 (31.9)		157 (46.0)	184 (54.0)	
Ethnicity				0.003			0.006			0.643
Non-Hispanic**	1506 (74.6)	68 (4.5	1438 (95.5)		978 (68.0)	460 (32.0)		201 (43.7)	259 (56.3)	
Hispanic**	393 (19.5)	19 (4.8)	374 (95.2)		284 (75.9)	90 (24.1)		42 (46.7)	48 (53.3)	
Unknown	121 (6.0)	14 (11.6)	107 (88.4)		80 (74.8)	27 (25.2)		14 (51.9)	13 (48.2)	
Race				<0.001			<0.001			0.005
White	975 (48.3)	26 (2.7)	949 (97.3)		631 (66.5)	318 (33.5)		154 (48.4)	164 (51.6)	
Black or African American	521 (25.8)	53 (10.2)	468 (89.8)		322 (68.8)	146 (31.2)		48 (32.9)	98 (67.1)	
Others		22 (4.2)	502 (95.8)		389 (77.5)	113 (22.5)		55 (48.7)	58 (51.3)	

**Table 2:** Results of the Electronic Outreach Program for CRC Screening CRC: colorectal cancer screening; PCP: primary care provider

Variable	N (%)
Total number of messages sent	2020
Total number with inactive electronic portal	101
Total number of patients with an active electronic portal	1919
Total number of patients who responded	577 (30.0)
Total number of responses	577
Request for orders for CRC screening	320 (55.5)
Declined screening	25 (4.3)
No longer patients of our clinic	28 (4.9)
Previously completed screening	135 (23.4)
Would defer to PCP or other specialists	51 (8.8)
Other responses	18 (0.03)
Request received for CRC screening orders	320
Orders placed for a colonoscopy	75 (23.4)
Orders for Cologuard	245 (76.6)
Request received for CRC screening orders	320
Completed screenings	176 (55.0)
Completed screenings	176
Colonoscopy	25 (14.2)
Cologuard	151 (85.8)
Positive Cologuard result	15 (9.9)

Regarding outcome measures, 176 out of the 320 orders placed (55%) resulted in the following completed studies: 25 colonoscopies and 151 mt-sDNA CRC stool tests (Cologuard). Patients were more likely to complete the mt-sDNA CRC stool test (61.6%) compared with colonoscopy (33.3%) (p<0.001) (Table 3). The positive mt-sDNA CRC stool test result rate was 15 out of 151 (9.9%), and none had CRC on subsequent colonoscopies.

**Table 3:** Screenings Completed by the Type of Colorectal Cancer Screening Method

	Completed screening		
Screening modality	Yes	No	
Colonoscopy (N=75)	25 (33.3)	50 (66.7)	<0.001
Cologuard test (N=245)	151 (61.6)	94 (38.4)	

Balance measures included an increase in in-basket messages; specifically, one of our faculty members received the majority of the incoming patient messages requesting orders for the CRC screening, which increased the time spent on in-basket messages. For those patients with a positive mt-sDNA CRC stool screening test, additional costs for a diagnostic colonoscopy were incurred, which were subject to the individual’s insurance costs. Lastly, patients expressed concern and dissatisfaction with the multiple reminder messages sent by the automated messaging system.

**Figure 1:**
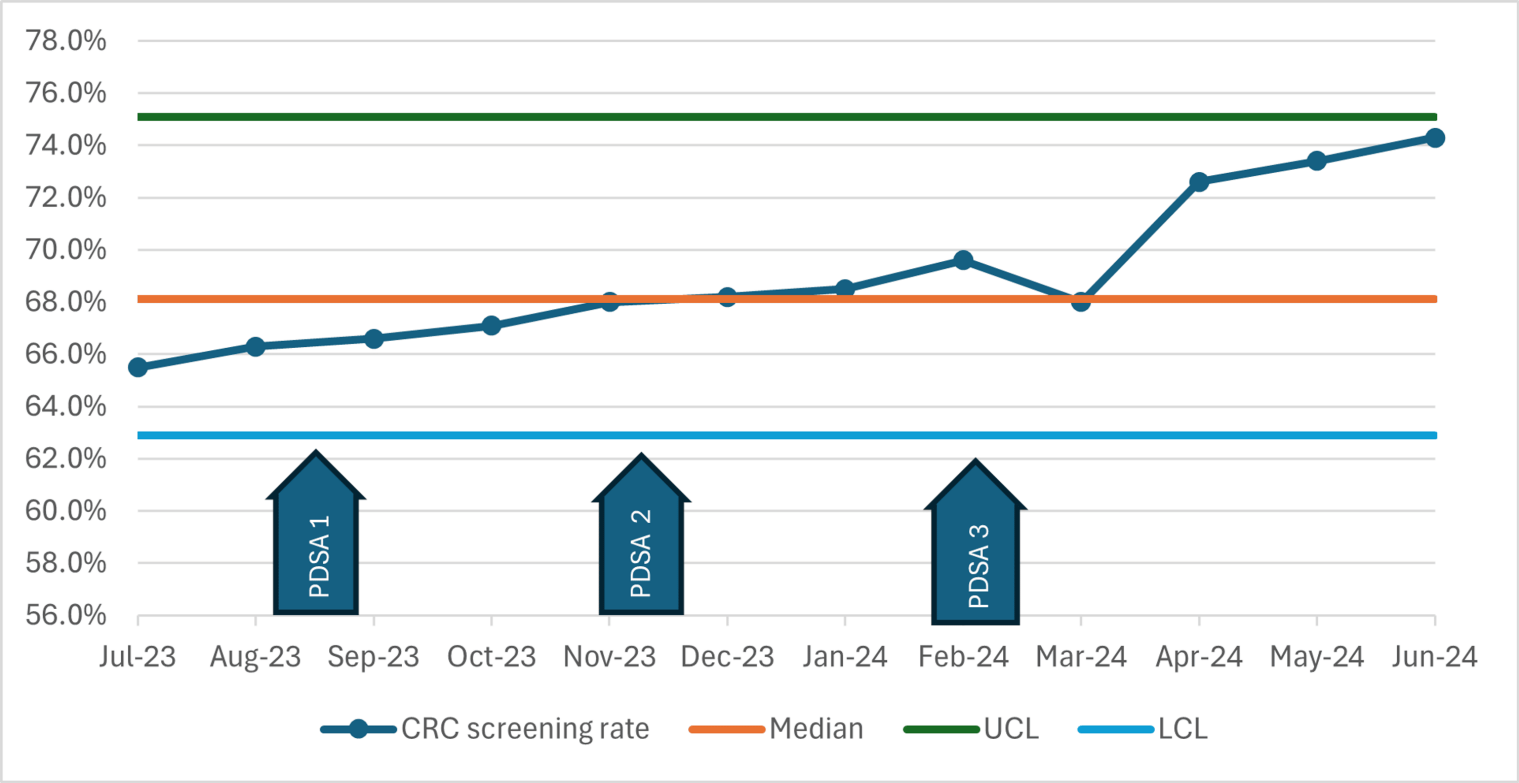
CRC Screening Rate Among Persons Aged Between 45 and 64 Years PDSA: Plan-Do-Study-Act; CRC: colorectal cancer; UCL: upper control limit; LCL: lower control limit

**Figure 2:**
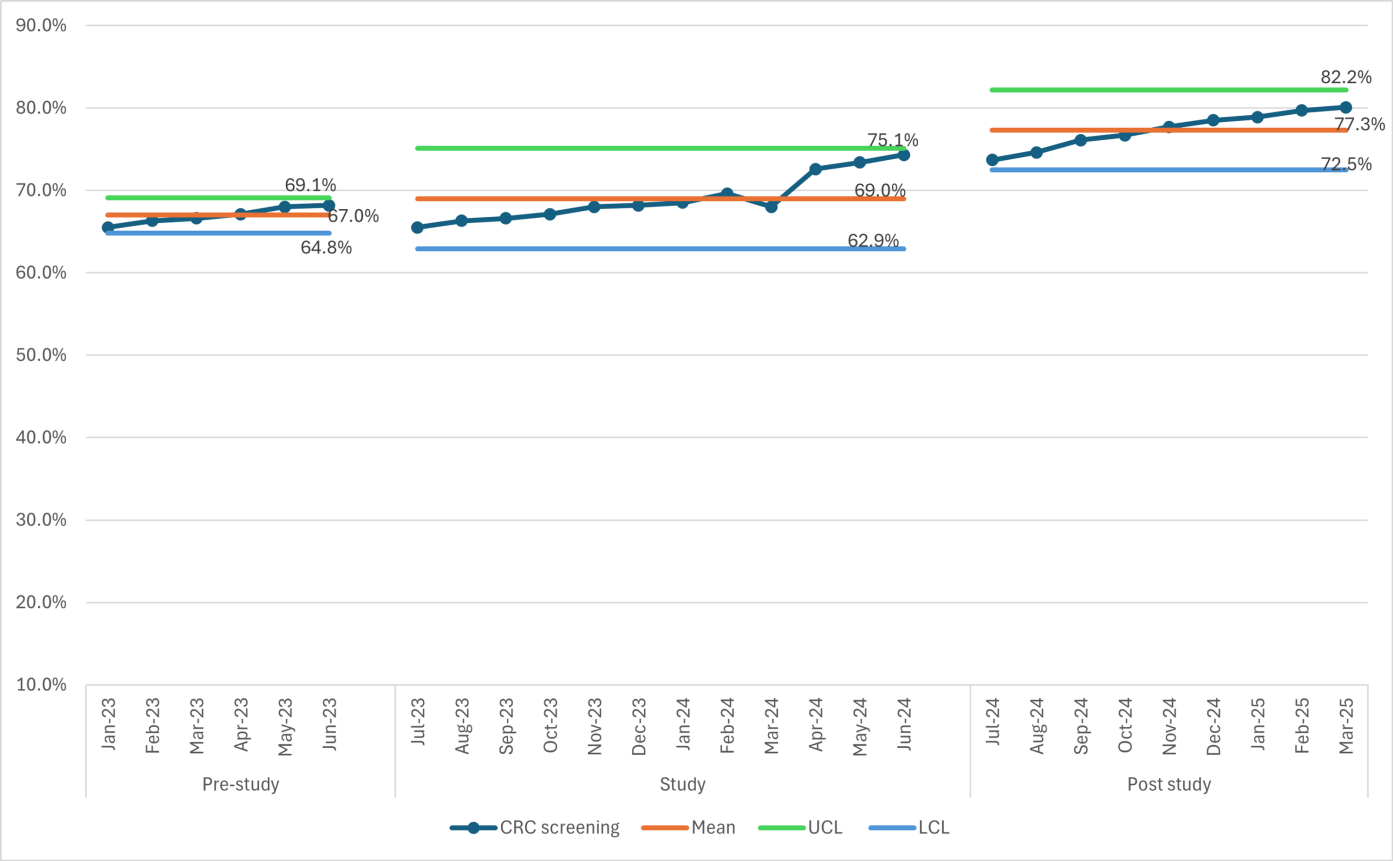
CRC Screening Rates: Monthly Statistical Process Control Chart (Pre-, During and Post study) CRC: colorectal cancer; UCL: upper control limit; LCL: lower control limit

Overall, CRC screening rates increased from 65.5% in July 2023 to 74.2% in June 2024 among patients aged 45 to 64 years old at our clinic (Figure 1). The greatest improvement was observed following the implementation of electronic messaging in PDSA cycle 3. Post project completion, despite this being a single outreach, the rates have continued to increase and reached 77.7% and 80% five months and nine months afterwards (in November 2024 and March 2025) (Figure 2), surpassing the initial 10% increase in goal, as more results became available.

## Discussion

During the implementation of this quality improvement project, CRC screening rates steadily increased from 65.5% to 74.2% during the study period (from July 2023 to June 2024) and to 77.7% and 80.1% five and nine months afterwards in our clinic's eligible patient population by using a multimodal approach including educational presentations for faculty to promote best practice and encourage screening orders, offering alternative screening methods for patients who declined colonoscopy, disseminating informational brochures to patients, and leveraging electronic patient portal messaging to offer at-home mt-sDNA CRC stool tests and colonoscopy. Although the data points remain within control limits, indicating a stable process, the gradual upward shift in rates, particularly after PDSA 3, may suggest a true process improvement rather than random variation.

The overall increase in screening adherence is similar to that of other studies showing that proactive, population-based outreach such as patient and provider education, automated phone calls, serial text reminders, and mail outreach can lead to improved screening rates [14-19]. We employed a multimodal approach, which also included serial electronic reminders at days 15, 30, and 45. Batarseh et al. also showed that using multi-faceted approaches may also be effective in improving CRC screening [14].

The use of serial electronic messaging through the patient portal proved to be a feasible method for reaching a large number of patients, with 95% of the target group having active accounts. A comparable study used serial text messaging, which, when combined with an opt-out mailed FIT kit, led to a 19.6% screening rate when compared with a control group (usual practice of a simple text message reminder) with only 2.3% success [15]. Similarly, another study showed that sending serial text messages also improved CRC screening [19]. In contrast to this, a study that used a one-time text message did not see a significant improvement when compared with the group that did not receive any text [16]. Although we saw an improvement in our CRC screening, our 30% patient response rate to the serial messages suggests that while the reach is broad, engagement remains a significant hurdle.

A key factor noted in our study was offering the at-home mt-sDNA CRC stool test, which was ordered for 245 of the 320 patients who responded. Patient preference for a noninvasive, at-home option overcoming the barriers to colonoscopy is consistent with some other studies [20-23]. Research from another health system showed that the adoption of stool-based testing correlated with a significant increase in overall screening adherence, rising from 26% to 49% over a five-year period as mt-sDNA CRC stool test use increased 40-fold. By reducing barriers such as transportation, time off work, and the need for bowel preparation associated with colonoscopy, at-home tests can improve uptake [21]. Our project further shows that offering different screening options, including less invasive tests, is a crucial strategy for boosting participation.

Despite the placement of 320 screening orders and automated reminders at 15, 30, and 45 days, only 55% of these resulted in a completed test. This gap between ordering and completion is a major challenge in many screening programs. This may be due to the lag time between the date of scheduling and the completion of the test. Other screening initiatives have also documented low participation rates; for example, a corporate-based program in Austria achieved only a 23% uptake despite facilitated email invitations and personal letters sent [24]. It was important to note that a few patients also expressed dissatisfaction with multiple automated messages; this showed the delicate balance between using convenient communication methods like this with the messaging frequency. Future interventions should refine messaging strategies to be effective without causing patient fatigue, an approach supported by "lead time messaging," which emphasizes planned, repetitive contact before screening is due. It was proposed by Jones (2020) to improve on-time screening by normalizing the conversation and providing adequate time for decision-making [25].

The clinical outcomes of the mt-sDNA CRC stool tests present another important area for discussion. The 9.9% positive rate was observed in our studies compared with other studies with rates ranging from 8% to over 20% in some other projects [14, 20, 22, 24]. However, the fact that all 15 positive results were followed by diagnostic colonoscopy without findings of colon cancer highlights the issue of possible false positives, which is a known limitation of stool-based testing. This outcome has implications, as it subjected 15 patients to the additional costs, risks, and anxiety of a diagnostic colonoscopy. This indicates the necessity for providers to engage in shared decision-making, clearly explaining the possibility of a false positive and the need for a follow-up colonoscopy before a patient chooses the mt-sDNA CRC stool test.

Finally, our outreach revealed issues with data completeness. Nearly a quarter (23.4%) of the patients who responded to our messages reported they had already completed screening, suggesting gaps in system documentation rather than non-adherence. This challenge is common in healthcare systems, particularly when care is obtained across different facilities, and it can lead to inefficient and potentially annoying outreach to patients who are already compliant. Similarly, another quality improvement project identified inaccurate data capture as a major barrier, prompting the creation of a dedicated population health dashboard to enable effective tracking and sharing of data with stakeholders [14]. This finding highlights the importance of robust, interoperable electronic registries to accurately track screening status and target outreach effectively [18].

Limitations

This project has several limitations. First, as a single-center quality improvement project and with a high patient portal adoption rate (95%), these findings may not be generalizable. Second, the absence of a control group makes it difficult to definitively attribute the entire increase in screening rates solely to our intervention. Third, given this was a descriptive analysis without statistical hypothesis testing, we could not determine if observed increases exceeded expected random variation or represent statistically significant improvements attributable to the intervention. Fourth, our multimodal intervention, which combined educational presentations to faculty, dissemination of informational brochures to patients, electronic messaging, and repeated automated reminders with a choice of screening modality, makes it impossible to determine the independent effect of each component on patient behavior. The data did not allow for disaggregation by race because some subgroup counts were too small, and this could mask disparities affecting these other minority races. Finally, the success of an outreach strategy that utilizes electronic messaging favored patients with access to the electronic portal over patients who do not use or have access to electronic messaging.

Additionally, we had about a 30% response rate to electronic messaging. This is similar to another study in which response rates to the secure email messages in an effort to increase CRC screening rates were approximately 23% [26]. The 70% non-response rate in our study introduces potential selection bias. Respondents may have been more health-engaged, better educated, or faced fewer barriers to screening than non-respondents. The intervention's effectiveness in harder-to-reach populations remains unknown. Collecting this information could have provided deeper insight into patient-specific barriers to participation, a common limitation in screening studies.

## Conclusions

CRC screening rates increased in one year among patients aged 45 to 64 years old in our outpatient clinic. During the implementation of this multimodal proactive outreach strategy, CRC screening rates increased. While temporal association suggests these interventions may have contributed to improvements, more rigorous study designs are needed to establish causation. The strong uptake of the mt-sDNA CRC stool test confirms the value of providing noninvasive choices to patients. In addition, the high false positive rate (100% of positive Cologuard tests) underscores the importance of informed consent and shared decision-making when offering stool-based screening, as patients must understand and accept the possibility of subsequent colonoscopy. Future work should focus on optimizing communication strategies and using multifaceted approaches to improve CRC screening.
